# What I Wish You Knew: Insights on Burnout, Inertia, Meltdown, and Shutdown From Autistic Youth

**DOI:** 10.3389/fpsyg.2021.741421

**Published:** 2021-11-03

**Authors:** Jasmine Phung, Melanie Penner, Clémentine Pirlot, Christie Welch

**Affiliations:** ^1^Department of Occupational Science and Occupational Therapy, Faculty of Medicine, University of Toronto, Toronto, ON, Canada; ^2^Bloorview Research Institute, Holland Bloorview Kids Rehabilitation Hospital, Toronto, ON, Canada

**Keywords:** autism, arousal regulation, insider perspectives in research, burnout, meltdown, shutdown, inertia

## Abstract

**Introduction:** Burnout, inertia, meltdown, and shutdown (BIMS) have been identified as important parts of some autistic people’s lives. This study builds on our previous work that offered early academic descriptions of these phenomena, based on the perspectives of autistic adults.

**Objectives:** This study aimed to explore the unique knowledge and insights of eight autistic children and youth to extend and refine our earlier description of burnout, *i*nertia, and meltdown, with additional exploration of shutdown. We also aimed to explore how these youth cope with these phenomena and what others around them do that make things better or worse, with a hope to glean knowledge to design better supports.

**Methods:** One-to-one interviews were conducted with eight children and youth, who shared their experience with BIMS. To match individual communication strengths of children and youth, we took a flexible approach to interviews, allowing for augmentative communication systems and use of visual images to support verbal interviews, as needed. We conducted a reflexive, inductive thematic analysis, using an iterative process of coding, collating, reviewing, and mapping themes.

**Findings:** Our analysis has identified that these youth describe BIMS as a multi-faceted experience involving emotional, cognitive and physical components. Moreover, these multifaceted experiences are often misunderstood by neurotypical adults, which contributes to inadequate support in managing BIMS. Of the four experiences, these youth identified meltdowns as most common.

**Conclusion:** By gaining first-hand perspectives, we have identified novel insights into BIMS and developed a more holistic understanding of these phenomena. These youths’ descriptions of supportive strategies for BIMS stress the importance of compassion and collaboration from trusted adults. This new knowledge will provide a foundation for how to better support autistic children and youth. Further research is required to develop an understanding of BIMS, especially with respect to how it is experienced by children and youth. Future research should leverage the insights and experiential knowledge of autistic children and youth to co-design support tool(s) for BIMS.

## Introduction

Autistic people who are engaged in public discourse on social media highlight burnout, inertia, meltdown, and shutdown (BIMS) as important parts of their lives (Welch et al., 2020b; Buckle et al., 2021; Higgins et al., 2021). Despite the high frequency and deep importance expressed by autistic people (Welch et al., 2020b; Buckle et al., 2021; Higgins et al., 2021), there is very little exploration of these phenomena in clinical literature. The literature is especially void of empirical explorations of these phenomena from the perspectives of autistic people themselves. For some notable exceptions, please see Raymaker et al. (2020), as well as Higgins et al. (2021) for explorations of burnout, Buckle et al. (2021) for an examination of inertia, and the work of Belek (2018), which explores meltdown and shutdown. Despite the strength of this early work, academic exploration of BIMS has been preliminary and these constructs are not yet clearly defined, delineated, or agreed upon. Our own work exploring BIMS has brought some of these phenomena to the attention of academic literature and offers only an early, somewhat tentative description of these phenomena, but leaves the need for deeper and broader exploration of autistic peoples’ experiences and understanding of BIMS phenomena. Additionally, our searches of the literature have not yielded any empirical explorations of these phenomena from the perspectives of children and youth. Table 1 shows a description of BIMS phenomena according to the current state of the evidence.

**TABLE 1:** Operational definitions of the manifestations of burnout, inertia, meltdown, and shutdown (BIMS) as described by autistic informants of earlier research.

**Burnout**	Described as a distinct source of severe and chronic exhaustion (Raymaker et al., 2020; Welch et al., 2020b). Autistic bloggers highlighted that the causes of this severe exhaustion are uniquely autistic such as “masking” – the constant need to exhibit appropriate behaviors to complete everyday tasks (Welch et al., 2020b). Informants have explained that this burnout often results in depletion of skills and intolerance to varying stimuli (Raymaker et al., 2020)

**Inertia**	A prolonged mental state of being “stuck” resulting in the physical inability to engage in activities that the individual wishes to do. Autistic individuals describe the experience of autistic inertia to vary in severity, duration of time and rate of repetition, however, all agree that when it does occur, it has the potential to be debilitating (Welch et al., 2020b)

**Meltdown**	A phenomenon with varying expressions by which autistic informants feel entirely overwhelmed accompanied by a lack of control and cumulative stress (Welch et al., 2020b). Meltdowns elicit responses of outward anxiety and energy outpour (Schaber, 2014). Some factors that contribute to a meltdown include, but are not limited to: social demands, frustration, embarrassment, challenges with communication, emotional triggers, and overwhelming aversive sensory stimuli (Welch et al., 2020b).

**Shutdown**	Although similar to meltdowns, shutdowns present as more internal experiences, where the individual withdraws from their surroundings and is accompanied by emotional pain (Belek, 2018). The degree to which one can function during a shutdown ranges from mild (e.g., being able to walk around and talk) to severe (e.g., feeling detached from your limbs and going into a fetal position) (Belek, 2018).

The lack of formal study of these phenomena translates to a lack of helpful strategies to support autistic children and youth with experiences of BIMS. Challenges with self-regulation (which we consider to be an important element of BIMS) are identified as a primary barrier to autistic children and youths’ success at school (Ghanouni et al., 2019). A support system or intervention approach is needed that can guide autistic children and youth, as well as the important adults in their lives, to effectively manage BIMS in ways that preserve the youth’s dignity, meet their individual needs, acknowledge strengths, and are informed by a neurodiversity framework (Milton and Moon, 2012; Leadbitter et al., 2021; Pearson and Rose, 2021). Also needed are new support systems and intervention approaches that support respectful communication and collaboration between autistic youth and their educators (Brownlow et al., 2021).

Our previous work (Welch et al., 2020b) began to address these concerns by generating an early academic description of burnout, inertia, and meltdown based on autistic insider perspective (see Table 1 for brief descriptions). However, this earlier work did not explore shutdown. Additionally, this work relied on analysis of blog posts and did not allow for further probing of relevant BIMS concepts, such as can be achieved through interviews. Finally, our earlier work did not explore the experiences of autistic children and youth, and so it is not known how or whether children experience BIMS, or whether they may experience the phenomena differently, nor is it known how best to support them.

We embarked on this study to build on our earlier work, to develop new knowledge of whether and how some autistic children and youth experience BIMS, and to draw on their experience, knowledge and insights to explore strategies for managing BIMS, thereby laying a foundation to design insider-informed supports and interventions. In this study, we pursued the following questions:

(Q1) How do these autistic children understand and describe experiences of burnout, inertia, meltdown, and shutdown (BIMS)?
(Q2) What can their experiences and insights teach us about supporting autistic children and youth with experiences of BIMS?

## Materials and Methods

### Context of Larger Study

This study takes place within a larger multi-phase research study. The larger study is called the Relax Recharge Ready (RRR) project and employs co-design methodology to better understand BIMS and to generate BIMS support tools that have been designed by and for autistic people. Both this study and the larger study operate within a constructivist paradigm, in which we acknowledge that every element of this research is influenced by the subjective experiences and assumptions we bring to the work (Annells, 1996). This article reports on an analysis of a subset of data generated within the larger study, specifically, data generated with our child and youth stakeholder group (ages 8–18). The first author (Phung) is particularly interested in child and adolescent development and took the lead in conducting a thematic analysis of the data that was generated with the children and youth participants of the RRR project.

### Ethical Considerations

A number of ethical considerations for research become especially critical for research that involves children (Crane and Broome, 2017). Of major importance, is the consideration of the power differential between researcher (an adult) and participant (a child/youth) (Crane and Broome, 2017). Autistic children and youth in particular, may enter the research process with previous experience of being disempowered when interacting with neurotypical adults (Lonbay et al., 2021). With this in mind, we gathered informed consent from both parents and children/youth prior to each interview. This included an explicit discussion of reasons a child or youth may wish to end the session as well as ways to indicate a preference to do so. We were also concerned that a child or youth may experience stress or distress during an interview, since this would be an unfamiliar experience with an unfamiliar person and focused on a topic that can evoke embarrassment and regret. A plan was created at the beginning of each interview to address and limit potential distress that could arise during the interview. Each interview began with questions such as “are there any signs that I can watch out for to know that you’re getting stressed and may need to take a break?,” “if you need help who can you call?” and “is there something that you love to talk about that makes you feel relaxed if you’re feeling a bit stressed?”. This plan provided a guide for the interviewer to observe for signs that the child/youth was distressed and what to do in that scenario. Parents were present while addressing the questions in the safety plan but did not have to stay for the entire interview unless requested by the participant. Of the eight participants, 1 participant requested for a parent to be present for the entire interview. Despite the risks of involving autistic children in youth in research, we believe that it is, on balance, more ethical to include them in the process (with measures in place to minimize potential for harm), than it is to exclude them from research that has the potential to impact their lives.

This study received ethics approval through the Bloorview Research Institute Ethics Review Board and through the Health Sciences Research Ethics Board at the University of Toronto.

### Participants

The child and youth participant subset included eight autistic children/youth. The youth were required to have a formal diagnosis of autism, be aware of their diagnosis (to avoid harm by accidentally informing a child/youth of their diagnosis during the research process), be 8–18 years old (median = 14) and able to communicate in English, either verbally or with a non-verbal communication system. Additionally, children and youth were expected to be able to discuss BIMS concepts (verbally or otherwise) as assessed by their parent. Demographic data was collected via a form that parents filled out prior to all interviews. According to the demographic data we collected, participants were from families of middle to high socioeconomic status and of Caucasian or European descent. Of the eight participants, five were reported as male and three as female. All participants also were reported to have an additional diagnosis: either attention deficit hyperactivity disorder (ADHD), anxiety disorder and/or an unspecified learning disability. No participants used alternative methods of communication. Some participants noted that they were currently taking medication and/or receiving therapy/intervention. Participants were recruited both internally and externally to the organization. We contacted individuals from our organization’s database of children and youth who are interested in research participation (via phone call to parents). We also sent out an information email via the list provided by a community partner. Additionally, a Tweet describing the study and with PI contact information was sent out from the media relations department of our organization.

### Positionality of Researchers

Our research team is made up of an early childhood educator/student occupational therapist (Phung), a developmental pediatrician and autism scientist (Penner), an autistic co-researcher (Pirlot) and an occupational therapist/occupational scientist (Welch). Our varied backgrounds influence everything about this work, from its inception to its implementation and its reporting (Annells, 1996). Immersion in occupational science and occupational therapy has made us particularly sensitive to elements of the youths’ accounts that relate to their daily occupations as well as the activity-based nature of strategies that the youth discuss. Despite the insistence that occupational therapy resists the “medical model” (Townsend and Polatajko, 2013), we recognize that we have been influenced by medical framings. Of particular note, the PI (Welch) initially approached BIMS phenomena with an “arousal regulation” lens, which was later revealed to be reductionistic compared to the descriptions given by the youth in this study. Another researcher (Penner) comes from a medical background and has clinical and research experience with autism diagnosis as well as multidisciplinary approaches to complex behaviors. The first author (Phung) conducted the analysis and therefore her perspectives and positionality are of particular importance. Phung approached this study with an appreciation for social emotional well-being in early childhood development, particularly the role of teachers fostering and nurturing this domain. In addition, Phung entered this study with the understanding that children have knowledge and insight of the world around them. However, the first author was cognizant of the previous assumption that autistic children may not be able to demonstrate the same metacognitive skills as neurotypical children. As this study progressed, this assumption has shifted into a greater appreciation of the unique ways autistic children and youth share their insight. Lastly, one researcher (Pirlot) has a background in sociology, as well as experience facilitating a support group for autistic people, whose experiences include BIMS phenomena. She is also an autistic advocate who used her unique position to inform the research, with a particular focus on ensuring autistic interests are represented.

As a team, we have approached this research with the shared assumptions that autism comes with unique insights and strengths and can also come with individualized challenges, especially when faced with disabling factors in the environment.

### Data Generation

The interviews were completed by the senior author (Welch). Each child/youth completed two interviews ranging from 25 to 45 mins long. We were committed to maximizing each child’s/youth’s unique communication strengths, and took a flexible approach to the interview process, as suggested by Teachman and Gibson (2013). The interviews occurred on Zoom and youth had the choice of keeping their cameras on or off to increase comfort during interviews. In line with the objectives of the study, interview questions were focused on BIMS phenomena, asking about participants’ experiences with each. Visual images were sometimes used to pull out additional details and descriptions, in a technique that had been informally piloted by Welch. The visual images were generated by a “Google Images Search” based on the interview questions and depicted children and adolescents in states of BIMS, according to the perception/interpretation of the interviewer. Of the eight youth, visual images were used with three of them. Participants identified relevant images that appeared through the search and the interviewer probed for better understanding of why images were selected by participants, see Table 2 for a description. The interviews were audio recorded and professionally transcribed, with the exception of one interview which was transcribed via Zoom (live captions). Transcripts were anonymized and stored on a secure server.

**TABLE 2:** Description of using visual images to support interviews.

**Step 1**	At the introduction to the interview, the child/youth was informed that the interviewer may show pictures about BIMS if it seems like it might be helpful to bring out ideas

**Step 2**	If during the interview, the interviewer detected challenges in getting verbal descriptions (e.g., participant answered with single-word answers, or gestures, or a response of “I don’t know”), the interviewer would share screen and show a Google images search of key words pertaining to the idea explored in the moment (child or teen plus: tantrum, yelling, feeling stuck, tired, and exhausted)

**Step 3**	Once the images were displayed, the interviewer would then ask: do any of these pictures show how this feels or what this is like? If the child says “no,” the interviewer conducts another image search

**Step 4**	When the child/youth selects an image that is deemed a good representation, the interviewer asks probing questions like – what do you think she is feeling? What do you think she is thinking? What might have happened just before? What might happen next?

### Data Analysis

Two research team members (Phung and Welch) met weekly to discuss the analytic process. A reflexive inductive analysis was used to facilitate a holistic understanding of these insider perspectives. A hallmark of inductive analysis is the process of generating codes based on data interpretations, rather than superimposing a list of codes generated prior to reading the data (Braun and Clarke, 2006, 2020). The analysis followed guidelines highlighted by Braun and Clarke (2006, 2020). Firstly, an open read of the data was completed and paired with analytic memoing to document reactions to the dataset (Braun and Clarke, 2006). The open read process allowed us to remain open minded to novel relationships in the data. Analytic memoing (conducted at all stages of the research) facilitated cognizance of our existing assumptions surrounding how we thought we understood BIMS phenomena (Braun and Clarke, 2020).

Through a second read of the data, initial codes were generated using Microsoft Word and Excel. Codes were then sorted by collating relevant data extracts and potential themes (Braun and Clarke, 2006). Thematic maps were created in order to visually represent the data and develop a better understanding of the relationships (Braun and Clarke, 2006).

**FIGURE 1:**
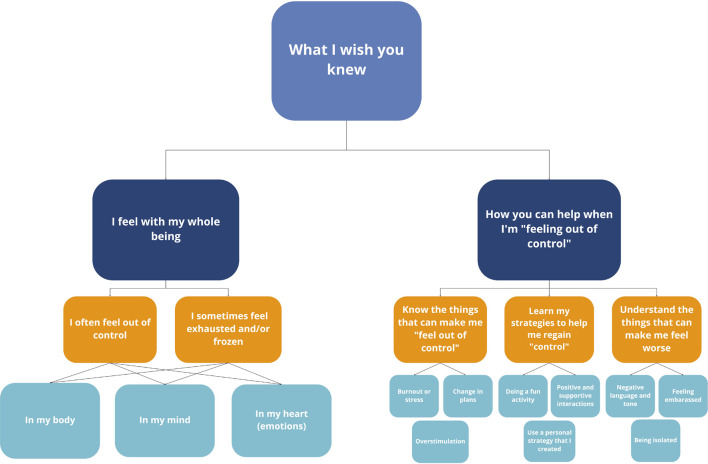
Text provided in quotation marks represent direct quotations from interviews.

Early themes were refined based on two criteria: internal homogeneity and external homogeneity (Braun and Clarke, 2006). Candidate themes were reviewed to ensure that collated extracts depicted coherent patterns (Braun and Clarke, 2006). Once candidate themes were refined, a series of thematic maps were created and further refined. We noticed that our names for BIMS phenomena were not generally used by the youth, with the exception of meltdown and therefore, renamed BIMS using the children and youth’s language and only used the BIMS terminology when applicable (see Table 3 for a translation of BIMS to the children and youths’ language). We have purposefully chosen to present the themes in first-person language to maintain the humanistic nature of these narratives. We also noticed that the children and youth used analogies to depict their experiences with BIMS. This was a key point in the analytic process and we decided to integrate the youths’ analogies to help structure our analysis. The data set was also revisited to ensure that the thematic map reflected the youth’s narratives (Braun and Clarke, 2006). Final themes were then identified and defined (see Figure 1).

**TABLE 3:** Renaming BIMS phenomena using the autistic children and youth’s language.

Burnout	Inertia	Meltdowns	Shutdowns
Feeling exhausted	Feeling stuck	Feeling out of control	Feeling frozen

### Steps to Ensure Rigor

Braun and Clarke (2020) provide a 15-point checklist and evaluation tool that was used to guide the quality of this data analysis. Our study places autistic insider perspectives at the forefront. This has been depicted through pivoting our language to match the participants, spontaneously generating codes based on narratives and the iterative process of analytic memoing (Braun and Clarke, 2006; Nowell et al., 2017). This aligns with the nature of reflexive thematic analysis as we have developed meaning and lessons through the collection of stories shared in combination to our unique positionalities (Braun and Clarke, 2020).

Our analysis, which is depicted through a thematic map, provides a visual representation of the relationships we identified between themes, sub-themes, and codes (Braun and Clarke, 2020). We revisited our early stages of coding and reflexive notes that described our early understandings of each theme prior to omitting. We also carefully assessed the relationships between chosen extracts and identified themes to ensure they were linked coherently (Braun and Clarke, 2006; Nowell et al., 2017). Analytic memoing was especially useful to track our justification of codes and themes identified (Nowell et al., 2017). To ensure dependability and confirmability (Nowell et al., 2017), an audit trail was created, retaining audio recordings, verbatim transcripts, documentation of coding process and theme/sub-theme identification, and analytic memoing/reflections.

## Findings

### Overarching Theme: What I Wish You Knew

During the interviews, the autistic children/youth shared their experiences with BIMS including what BIMS means to them, how they experience BIMS and helpful/unhelpful strategies they have implemented to cope with BIMS. An overarching theme was constructed: *What I Wish You Knew.* For these children and youth, they emphasized the importance of adults actively listening to their narratives to better understand their experiences with BIMS. They acknowledge that neurotypical individuals may experience BIMS to some degree, however, for these autistic children/youth, they acknowledge that they experience it *differently.* Within this overarching theme there are two themes: (1) *I feel with my whole being* and (2) *How you can help when I’m “feeling out of control*.*”* These two themes further consist of sub-themes which are represented by codes (see Figure 1).

#### Theme 1: I Feel With My Whole Being

The participants in this study provided descriptive narratives of their experiences with having meltdowns or “feeling out of control.” However, when discussing burnout, inertia, and shutdown, only some participants identified that they experience these. For those who provided descriptions for burnout, inertia, and shutdown, overlapping and synonymous descriptions were used when discussing each phenomena. In order to remain in alignment with the children and youth’s narratives, burnout, inertia and shutdown were grouped together using the participants’ language: “feeling exhausted” for burnout and “feeling frozen” for shutdown and inertia.

The children and youth utilized analogies to depict their experiences with “feeling out of control” and feeling “exhausted and/or frozen.” These analogies elucidate that the aforementioned phenomena are multifaceted experiences that include emotional, physical and cognitive components. These multifaceted components are represented through three codes: *in my body, in my mind*, and *in my heart.* Passages in the interviews that described physical sensations, bodily reactions or behaviors were coded as *in my body* and passages that highlighted cognitive processing, thoughts or beliefs were coded as *in my mind*. Responses that emphasized an emotional experience such as feeling overwhelmed, helpless, frustration or shame were coded as *in my heart*. Together, these multifaceted components depict how these phenomena are experiences that include the children/youth’s whole being.

##### Subtheme 1: I often “feel out of control” (meltdowns)

One child/youth (CY) presented the follow analogy for “feeling out of control” (meltdowns):

“You’re a passenger on a ride of destruction… and it’s like hitting a bunch of stuff.” – CY 6

*In my body:* The participants describe the physical experience of “feeling out of control” as consisting of an array of strong bodily reactions reminiscent of a physiological fight or flight response. They describe their bodies experiencing the following:

“My vision getting blurry” – CY 6

“Getting tense muscles and I start to get hot.” – CY 3

“My cheeks get warm.” – CY 4

“My hair sticks up.” – CY 4

“Shoulders bunch up.” – CY 2

“Yeah, I started yelling and, like, stuff and then like… yeah, I was like I was yelling and yelling and yelling a lot.” – CY 2

“My face gets a little bit red.” – CY 3

“I breathe fast.” – CY 2

“My sight is a little bit more restricted, like, I’d only really be able to see the room that I’m in and nothing else.” – CY 6

“I’ll just kinda hold my head from stress.” – CY 1

*CY 8:* It feels like I have a bit more power.

*Interviewer* (*I*)*:* Oh. Is that something you can feel in your muscles?

*CY 8:* Yeah. [indistinct] get an adrenaline rush.

*In my mind:* The thoughts that arise during these physical experiences further illustrate the symbolism of the passenger in the analogy. The participants’ cognitive descriptions evoke ideas of “feeling out of control”:

“It’s like my subconscious is still out of my control.” – CY 1

“… forcing myself to stay calm.” – CY 1

“It’s like tunnel vision.” – CY 5

“I don’t really remember what happens very well, it’s usually a little fuzzy.” – CY 5

“Feeling out of control.” – CY 5

“Like when I’m stressed, I’ll kinda stop and just get really stressed out while trying not to have an outburst. Or I’ll actually manage to stay calm” – CY 1

“When I’m in a meltdown it’s not easy to get out of and I’m not in full control, like I’m not thinking clearly.” – CY 5

“A by-product of my stress.” – CY 1

*In my heart:* Along with the strong embodied reactions and “tunnel vision,” the participants also placed emphasis on the high emotions associated with “feeling out of control.” The occurrence of these components reflect intrusiveness to their daily tasks. They stated that despite their efforts to maintain composure, high emotions can build up resulting in stress and frustration, and overall feelings of helplessness and reduced motivation to participate in other activities. The participants stated:

“What happens when I’m trying to be calm is like, just the stress will just continue building up, that’s usually what happens in anything is like, whenever I try to contain any sort of emotion or anything, it’ll just keep building up, usually with stress or anger it happens the most where it will just keep on.” – CY 1

“It’s kind of like there’s just no other thoughts than just anger.” – CY 6

“You feel a different emotion every time a meltdown ends, depending on what it started from.” – CY 7

“It’s gotten to the point where I’m so unmotivated that I can’t even do things that I want to do.” – CY 1

[Looking at pictures generated by Google Images]

*I:* Hmm, what kind of picture should I look for?

*CY 7:* Angry.

… It has the face, it has the face, just pretend that, like, 10,000 [indistinct] just make that face angrier.

##### Subtheme 2: I sometimes feel exhausted and/or frozen (burnout, inertia, and shutdown)

*In my body:* When discussing their experiences with feeling “exhausted” and being in a “frozen” like state, participants described similar physical manifestations of each. One youth presented the following analogy for feeling exhausted (burnout):

“You can have this really really big dough and it’s really hard and tense” – CY 3

This participant’s analogy represents the physical effort to knead a tense and hard piece of dough. The continuous effort to knead the dough results in eventual fatigue in our arms. When sharing their experiences with burnout, the youth describe similar physical feelings as depicted in this analogy. They placed emphasis on physical exhaustion when required to complete tasks throughout their daily life such as school work. They stated:

“Sometimes that happens to me, like, when I come home from school after I’ve had, like, two tests and a ton of assignments. I just come home and then I just sit at my computer for a few minutes not knowing what to do.” – CY 6

When feeling “frozen,” some participants provided the following analogies that also depict physical tiredness and a physical internal conflict that they are trying to overcome. The analogies are provided, respectively:

“It kind of feels like my blanket weighs 500 pounds and it’s weighing me down.” – CY 6

“… a slow old computer that’s trying to run Google Chrome… it just uses up a lot of RAM.” – CY 1

The participants use an “old computer” and a “heavy blanket” to represent a combination of feelings: decreased physical energy, lagging, slowness, and being physically stuck. Specifically for shutdowns, one youth identified that their physical tiredness can occur when they are feeling overloaded by environmental stimuli:

“Just feeling drained, like I don’t have the energy to get up.” – CY 5

“Lazy. Tired. Maybe even a little bit of exhaustion. In my whole body. I feel weak, not… like my body is heavy.” – CY 5

“Something that is difficult to get out of.” – CY 2

“You’re frozen and you can’t really get to that thing.” – CY 3

“yeah sensory overload I mean it doesn’t happen that often but like with ASD it does definitely happen.” – CY 8

*In my mind:* The participants described the occurrence of cognitive difficulty when overcoming their physical manifestations of “exhaustion” and “frozenness” when balancing a number of responsibilities. These cognitive experiences can occur when having to decide between a number of choices, specifically with completing school work (e.g., either choosing to take a break or to persevere through the task). They highlight this cognitive experience as stressful and hard to overcome, stating that:

“It’s more like artist’s block or writer’s block when it happens to me” – CY 6

“I think I should be able to do this on my own, I don’t really want to ask for help but I’m still stuck, and I don’t know how to do this. It would be great if I asked for help, but I don’t really want to ask for help. It’s this hard.” – CY 3

“What happens to me is if there is a lot going on like my family arguing and I have to finish my work, it just gets really hard and I need a break but I also feel like I have to do the work.” – CY 3

“It feels like my mind is struggling to do anything.” – CY 1

“Shutdown is just, like, usually ‘cause of stress, I’ll just completely freeze up, I can’t really talk, I’ll stutter, I’ll like literally freeze up. I’ll sit down or something, I’ll make weird noises that’s basically just the sound of my mind straining to work.” – CY 1

Specifically for inertia, one participant identified a fine difference from burnout and shutdown. This participant described inertia as having difficulty with task initiation resulting in challenges with productivity. They stated:

“Having trouble getting started with something.” – CY 8

“Trouble getting the ball rolling, actually getting to work on something.” – CY 8

*In my heart:* When discussing “exhaustion,” participants identified feeling relief when the “exhaustion” passes. One youth recounts:

“I feel good when it’s done, but when it’s going I get a little stressed out because it’s going too fast for me. When you mentioned that point, for me, I think it’s like climbing walls and if I do one, and if I finish one, that’s just really really high, I’m happy, but I’m also a little bit scared for the next one that they want me to do.” – CY 3

When discussing “feeling frozen,” their narratives also highlighted a relationship between their emotional experiences and physical/cognitive components. Participants identified feeling frustrated because they recognized their responsibility to fulfill tasks demands but are unable to. A participant describes their experience of needing to complete a 3-page essay:

“And so I’ve been working on, like, just putting it onto a page, right? My Mother is helping me write, like, a [indistinct], but currently I still feel as [indistinct] I’m like okay, but I have all the ideas, why can’t I just submit this, like, why do I now have to put it all into a 3-page essay, this is going to be a pain, right?” – CY 8

Another participant also used an analogy to represent two feelings and the cognitive dilemma that they experience when “feeling frozen.” Importantly, this quote also demonstrates that these phenomena can evoke a spectrum of experiences (e.g., “feeling frozen” can evoke both an emotional and cognitive experience) rather than each occurring in isolation. This youth recounts this feeling at school:

“Uh-huh. I… it… it always, usually it just happens to me when I get angry, or I get really upset, or like… it happ… like, I have those two emotions at school and it’s… and, like, a friend did something that I didn’t really like, and it’s just like I want to say the things at them, but it was… it was sort of like tug of war, one side wanted to, like, stomp at them and say mean things at them; and then the other side didn’t know what, and then the other sides didn’t want to because, like, it’s my friends and I should be nice to my friends, and sometimes when that tug of war thing happens, I get stuck. And I don’t know what to do, and I want to do some… and I want to shout mean things at a friend, but I feel like I can’t.” – CY 3

#### Theme 2: How You Can Help When I’m “Feeling Out of Control”

Of the four BIMS phenomena, the participants explicitly identified meltdowns as most prominent in their lives. This may be reflective of their social environments (e.g., topic is often discussed amongst adults), the intrusiveness to their daily lives, development of vocabulary to allowing richer discussion of meltdowns, or a possible developmental/maturational component. When discussing meltdowns, they highlighted three main ideas: Know the things that can make me “feel out of control,” learn my strategies to help me regain “control” and understand the things that can make me feel worse.

##### Subtheme 1: Know the things that can make me “feel out of control”

*Feeling exhausted* (*burnout*) *or stressed:* When asked about their experiences with meltdowns, the participants shared instances that led to a meltdown. Their descriptions highlighted that a build-up of burnout and stress, and feeling drained from an accumulation of task demands, may lead to experiencing a meltdown. This indicates that these participants may experience burnout and meltdowns simultaneously. These participants recount:

“I was all… a little bit mad, at first, but I was a little… really tired, but then when they got back to school, the education assistant (EA) was constantly bugging me “do the work, do the work, do this, do this work…” I don’t remember what it was but it was pretty hard work. “Do the work, do the work, do the work.” I was like “No, I’m extreme… I have no energy left, like, and it wasn’t like how I normally say I get tired just to kinda [indistinct] like just to kinda, like… like usually when I say I’m tired I’m, like, a little bit tired but this… like, just [indistinct] no, I was genuinely for once actually too tired to do any work, like, my brain was just… falling asleep. I was, like “please no, I’m extremely tired, like, I’m not even joking.” And he was like “no, no no, you’re going to do the work” like, “No, I’m tired. And I was getting more agitated.” – CY 1

[Looking at pictures generated by Google Images] “She did too much… she did too much work and then she needed to do more.” – CY 4

“Like if a whole bunch of minor things that I’m not in the mood and they are they just keep on stacking up.” – CY 8

*Unexpected change in plans:* Moreover, some participants describe that an unexpected change in their plans like planning to play a specific game during recess but suddenly unable to, can also result in a meltdown:

“Actually usually it’s more because something, like really goes off of your plan, like, it’s not that you don’t get what you want, like, if you say that you want to play with a ball during recess or something, then someone else takes the ball an hour before recess, that’s just plain rude, even if you, specifically told them that you want to play with it.” – CY 6

*Overstimulation:* Lastly, the participants identified that over-stimulation (sensory/social/cognitive) can result in them feeling out of control:

“That’s the best way I can describe it. Yeah, has [indistinct] fun, and of course I got stressed out because lots of stimulation.” – CY 1

“But for me it’s just, like, really high pitch, like, when someone’s like scraping a fork and knife together or something.” – CY 6

##### Subtheme 2: Learn my strategies to help me regain “control”

The youth provided descriptions that adults advised they try, as strategies to help when *losing control* (see Table 4 for summary of strategies). However, this participant states that at times these general methods are not as useful as we think:

“Well, something that every adult that I’ve ever talked to about this kind of stuff tells me – just walk away. Except it’s not very helpful advice because sometimes it isn’t possible to walk away, sometimes you’re in class and you can’t walk away, you’re stuck there for the rest of the day.” – CY 6

**TABLE 4:** Summary of strategies for meltdowns.

Fun activities	Positive/supportive interactions	Other strategies
Playing with Play-Doh	Sitting with family and friends	Setting a timer on iPad
Doing an outdoor activity	Talking to family and friends about interests	
Listening to podcasts or songs	Hugging/cuddling with pets or stuffed toys	Mindfulness based strategies such as taking deep breaths
Playing sports such as karate and soccer	Stepping away from others or being alone momentarily	Counting to 100 with eyes closed
		Distraction techniques through use of imagination and visualization
		Devise response/support plan based on individual preferences: touch/no touch, talk/no talk, eye contact/no eye contact

*Fun activities:* In contrast, the participants identified strategies that they have used and actually found helpful in regaining control of their thoughts, physical reactions and emotions. During discussion of each participant’s safety plan, some participants identified that talking about their interests (e.g., dogs, science, and space) helped to calm themselves down. Moreover, many of the participants identified that doing fun activities was also a helpful strategy. A participant noted that doing fun activities like soccer, has allowed them to healthily cope with strong emotions:

“Playing with Play-Doh” – CY 3

“Doing a fun outdoor activity” – CY 3

“Two things that helps me is (1) curling into a ball, and (2) listening to new podcasts or songs that I like in the headphones” – CY 3

“In third grade I would definitely, that’s definitely an aggressive kid. Before, but then I started playing karate actually” – CY 8

“When I get frustrated, usually I want to kick things and throw things, but I also know that it’s not a good idea … well, I’ve been thinking about a way to not hurt anybody, well, it’s kicking a soccer ball because it’s fun and I usually compete against a wall and you can kick it really really hard, like, you can kick it with all your anger and it could be… it could be helpful sometimes.” – CY 3

*Positive/supportive interactions:* From the participants’ descriptions, another type of strategy was identified: positive/supportive interactions with family, friends and pets. They highlight that talking through their thoughts and emotions during a meltdown can be helpful in calming them down:

“Yeah. Well, I think one strategy, the first strategy that I… that I think I can do, if it’s with my cousins, my sister and my Mom have been telling me if you get upset, talk, and I’m unavailable, you can talk to your sister, and then if I’m suddenly available and your sister can talk to me.” – CY 3

“Vali is the solution to all of my problems. Well, yeah, she’s kinda meant to, she’s trained to, she’s a service dog.” – CY 1

Some of the participants also emphasized the importance of listening to what they have to say during a meltdown:

*I:* So obviously what people say when someone’s in a meltdown is important, so what would be a good thing to say?

*CY 1:* A good thing to say would be nothing, and to let me talk.

“I liked talking about games” – CY 3

They also noted that at times, physical touch from their loved ones provide a sense of comfort and reassurance:

“Giving me a hug” – CY 3

“Play with my dog” – CY 6

“Often cuddling my mom, and cuddling Wedgie often helps” – CY 5

Instead of receiving physical comfort, other participants discussed having a “body break” or removing themselves from the current social/physical setting, allowing them time and space to calm down:

“Run away … run under my bed.” – CY 3

“I feel what’s helpful for me is just being alone.” – CY 1

*Strategies I’ve learned:* The third type of strategy that the youth discussed was the internalizing and adapting formal strategies taught by adults. For example, one participant discussed the use of an iPad to help manage their behavior:

“Because I use it so much that, I thought I should probably try setting all my timers on my iPad because I actually pay attention to it, because it’s like the… one of the few things I actually consistently will pay attention to” – CY 1

Other participants discussed breathing techniques, recounting helpful mindfulness strategies that they previously learned.

“Like taking deep breaths” – CY 2

“Sometimes I just sit there, close my eyes and count to 100, and then… and then open them again” – CY 6

Lastly, the participants identified the use of imagination to think about happy things or using distraction techniques like visualization:

“Kind of imagine yourself in your happy place, but, like, imagine that you put on a VR headset and you’re, like, wherever you want to be.” – CY 6

“Imagine a cat or a dog just, like, standing around you and just, like, brushing up against you sometimes makes you feel calmer because you feel like there’s someone there with you kind of protecting you in a way” – CY 6

“Something that I used to do, and I don’t… it’s probably really uncommon, but it could be on your list of strategies was this thing I did where I imagined that there’s, like, this guy on a motorcycle or he was running or something, and just all of the little ledges on the wall he could… and, like, the lines he could run on. So I made a whole course for him in different rooms.” – CY 6

##### Subtheme 3: Understand the things that can make me feel worse

The participants provided insightful responses regarding behaviors and interactions that have exacerbated feeling out of control, magnifying the stress of their experience. They highlighted the impact of communication, specifically the type of language and tone used when speaking with a child who is already feeling overwhelmed and upset:

“I don’t know why, but this one EA gets [indistinct], but she was telling me to do my work, and I was like no, I genuinely am burnt out, tired, I can’t. And then… I’m… like I don’t have the full memory, but I know that, like, she genuinely insulted me, like, by telling me like you’re acting like a little kid, you’re acting like… like she said that over and over. Like she was trying to provoke me or something. And I just lost it.” – CY 1

“Like, if some… if I’m really… if I’m really really upset and it’s not a person like my Mom, I… and if they’re just saying, like, stop, that usually… it doesn’t really help. And if they’re saying it in, like, a mean sort of tone of voice, that makes it even worse, and that you add, and like, add sad to the bucket of negative emotions that I’m feeling in that moment.” – CY 3

The participants have identified the value of social support from teachers, principals, family and friends, and further associate feelings of embarrassment and shame when isolated or when a space is being evacuated during a meltdown. They wished that teachers and other supportive adults knew how to support them during a meltdown:

“It felt pretty embarrassing because, like, I was the reason that the class couldn’t learn, and… that’s pretty much all I really felt.” – CY 6

“For me it’s just I hate being alone. Like… I know they’re right outside the door, ‘cause they’re literally barricading me in.” – CY 5

“… I was having a meltdown at school, and… and the teacher just got the class to leave the room and that was it, they didn’t do anything else. They called the principal and then the principal came and… and then the principal calmed me down, the teacher just got the class out of the room and then called the principal” – CY 6

“Yeah. That’s why it kind of bugs me when the teacher just calls the principal, because actually now I have a pretty good relationship with the principal because I’ve been sent up to the office too many times to count.” – CY 6

## Discussion

This study offers a new understanding of BIMS as experienced by some children and youth. The findings in this study support earlier work (Belek, 2018; Raymaker et al., 2020; Welch et al., 2020a; Buckle et al., 2021) that BIMS is an important part of life for many autistic people. The descriptions offered by these children in many ways align and in some ways differ from descriptions provided by autistic adults who have informed earlier literature.

Raymaker et al. (2020) used interviews as well as internet sources to describe burnout from the perspectives of autistic adults. Like the informants in Raymaker’s study, the children and youth described burnout in terms of chronic tiredness, decrease in skills, and reduced tolerance to stimulation. Also similar, the youth in our study linked experiences of burnout to sustained high demands. Interestingly, the informants in the Raymaker study also linked burnout depletive effects of masking, whereas the children and youth in this study did not explicitly discuss masking (though admittedly, there were no interview questions pertaining to masking).

Our findings align with Buckle et al. (2021) examination of inertia which explored the experience of 32 autistic adults. Specifically both studies generated descriptions of inertia that encompassed physical, emotional, and cognitive factors. This is an important consideration in future work aimed at addressing inertia, as it signals a need to avoid over simplifying or reducing inertia to an issue explained only by motoric skills, executive functioning or emotional circumstance. Rather, these studies call for holistic and multifaceted approaches to understanding and supporting inertia.

Belek (2018) explored embodied experiences of autism that touched on both meltdown and shutdown. Belek’s description of shutdown emphasizes triggers like sensory overload and internal experiences of fear and paralysis that match descriptions from these children and youth. Belek’s description of meltdown also bears similarity to those offered by these children and youth in that they involve “feeling out of control” and can be terrifying. While one of our participants described a meltdown like being “a passenger on a ride of destruction,” one of Belek’s informants described meltdown as “a jet plane crash” (37). The children and youth in our study placed much more emphasis on meltdown than on shutdown as disruptive factors in their lives, whereas this weighting is not observed in accounts from adults (Belek, 2018; Welch et al., 2020a). It warrants further study to determine whether this represents a maturational effect in which autistic individuals learn to manage or avoid meltdowns, either through alternate strategies or through replacing meltdown with shutdown, as shutdowns often have fewer or milder negative consequences compared to meltdowns. As a participant in Belek’s study states, “I normally go in blank shutdown mode to control my meltdown”(36).

When compared to descriptions from our earlier work (Welch et al., 2020a), which was based on adult perspectives, descriptions of BIMS phenomena as provided by these children and youth use different terminology and are less clearly delineated, with some descriptions across phenomena being similar (e.g., the physical manifestations of burnout, inertia, and shutdown). The children also described a sometimes cyclical nature of BIMS phenomena, with partial overlap; for instance, burnout leading to meltdown or having a meltdown right at the beginning of a burnout. As noted earlier, differences in the child and youth descriptions compared to adult descriptions may represent developmental or maturational differences in how BIMS is experienced or expressed. It is also possible that the children and youth in our study have had less exposure to the concepts of burnout, inertia, and shutdown (though all were familiar with meltdown) compared to adults and therefore have less fully developed conceptualizations and vocabulary for these phenomena. The children and youth’s familiarity with meltdown terminology may reflect that it is one term often applied to neurotypical children, whereas burnout, inertia and shutdown are terms that come from the autistic community (Welch et al., 2020b).

### Implications for Practice

The descriptions from these children and youth illustrate that they experience BIMS in ways that are physical, cognitive and emotional. Their whole-person descriptions stand in contrast to the more reductionist theorizations of dysregulation and behavioral responses that are often seen in current literature and clinical approaches. Current clinical approaches tend to focus on executive function, physiology or behavioral responses in isolation (Hess et al., 2008). Approaches such as auditory integration training, sensory integration, and cognitive behavioral modification, aim to change autistic children’s intrinsic traits in order for them to better fit into their environments (Hess et al., 2008). Instead, the descriptions from the children and youth in our study support Leadbitter et al. (2021) suggestion that a crucial element of advancing autistic research and clinical practice is to understand autistic experiences as a way of being which is complex and dynamic.

The variations across the descriptions from these children and youth highlight the highly individual way in which autistic people experience and respond to things and alert us to the dangers of adopting BIMS as a reductionistic or essentialist way of explaining autistic experience. Rather than advancing a reductionist model, it is our hope that readers use these descriptions of BIMS to expand their thinking in relation to what autistic children and youth experience in daily life and approach the outward manifestations of BIMS, which are sometimes interpreted as laziness, resistant or avoidant behavior, and aggression, with curiosity, compassion and a spirit of collaboration.

Although our study highlighted a number of strategies that are helpful during “out of control” experiences (e.g., meltdown), it is important to remember that these strategies are highly individualized. Some of the youth in our study shared that they prefer physical touch from their loved ones during “out of control” experiences, as it provides security and comfort. However, other youth noted that they prefer to be left alone, which relieves them of demands like making eye contact and maintaining conversation (which makes meltdown and shutdown worse). Iemmi et al. (2017) emphasize that due to the variable presentation of autism, it is necessary that individualized strategies are differentiated by a variety of factors including individual preferences, age, gender and those with multiple diagnoses. Taking time to consider these factors and consult with autistic children and youth about approaches that are helpful to them will facilitate quality, individualized support that best aligns with each individual’s needs (National Autistic Taskforce, 2019). Lastly, many of the strategies shared by participants are not exclusive to autistic children and youth. These strategies could also be helpful to non-autistic peers and may fit well into a universal design approach within classrooms.

The thoughtful and actionable insights offered in these descriptions emphasize how autistic youth are the true experts in their own experiences. Some of the strategies highlighted by the youth, such as deep breathing and using cognitive strategies (e.g., visualization), align with conventional clinical approaches (Robert et al., 2013). Some of the strategies they have found helpful, such as supportive interactions and engaging in fun activities, are outside conventional clinical approaches. This is similar to findings from Pavlopoulou (2020) who elicited personalized accounts of effective sleep strategies from 54 autistic adolescents. Like the strategies described by the youth in our study, the successful sleep strategies identified sometimes aligned with conventional wisdom pertaining to sleep (e.g., relaxation before going to sleep), but sometimes diverged (e.g., control over sensory stimuli at bedtime). Both studies highlight the importance of looking beyond neurotypical-informed conventions when collaborating with autistic youth, as this can generate novel approaches for support.

The children and youth in this study express an awareness that most adults in their lives do not understand their experiences of BIMS. To help us understand, the youth frequently employed analogy and metaphor; drawing on something they believed us to know and then making connections to their own experience. This challenges the assumption that all children and youth identified as autistic will struggle with theory of mind or use of metaphor, as is sometimes reflected in the literature (Norbury, 2005), but aligns with findings from Olofson et al. (2014). Our finding that these children and youth do not feel that the adults in their lives understand BIMS, or collaborate with them to generate solutions, aligns with work from Brownlow et al. (2021), who found that autistic youth in classrooms felt that they were not included in informed decisions about their learning. Brownlow et al. (2021) recommend positive communication strategies to foster collaboration and positive relationships between students and teachers, something our data supports.

The children and youth in this study placed very high value on compassionate support and understanding from the adults around them. The youth who described the greatest success in their current management of BIMS described situations in which they had generated and implemented strategies through collaboration with an important adult (usually a parent or education aide). This finding supports a shift in the direction toward something we like to call “collaborative regulation.” Collaborative regulation could be seen as similar to a co-regulation approach [e.g., as described by Gulsrud et al. (2010), which used mother-mediated joint attention to support emotional regulation in autistic children], in that it acknowledges the influence of others on an individual’s level of arousal; however, collaborative regulation goes beyond co-regulation to acknowledge a shared responsibility for monitoring and supporting a person’s state of arousal. Additionally, a collaborative regulation approach, as we would like to put forth, emphasizes mindful and deliberate planning to set an individual up for success and includes consideration for the physical, sensory and social environment.

Collaborative regulation can facilitate opportunities to provide positive support and in turn, reduce feelings of humiliation, regret, and fear (Ting and Weiss, 2017). Adults can work together with autistic youth to scaffold useful strategies (Ting and Weiss, 2017) when they are feeling exhausted, out of control or frozen. Scaffolding includes sensitivity toward children’s emotions, providing encouragement and validation, and valuing children’s active participation in goal achievement (Hoffman et al., 2006). Buckle et al. (2021) identified that some autistic informants depend on scaffolding from their external environment when overcoming inertia (e.g., completing a task side by side with another individual) as it provides visual prompting, which further facilitates task participation and follow-through. Therefore, through collaborative regulation, autistic youth and teachers can determine together when and how to best apply scaffolding techniques in the classroom.

The youths’ emphasis on collaborating with adults for successful management of BIMS is interesting in that it has the potential to address the “double empathy” problem identified by Milton (2012) which suggests that empathetic disconnect between a neurodivergent person and a neurotypical person could improve through mutual and reciprocal efforts from both parties. The recommendations from these youth also align with the social model of disability (Oliver, 2013) in that many of their strategies are designed to remove certain disabling factors that cause or exacerbate BIMS. When discussing helpful strategies for coping with BIMS, the children and youth in this study do not advise hiding, covering up or masking the fact that they are struggling in a certain situation. Rather, they recommend open communication, interaction and collaboration to manage BIMS. Thus, collaborative regulation, individualized coping strategies and the unconventional approaches that were previously identified, are stepping stones for practical approaches that humanize autistic youth’s overall experience with the BIMS phenomena.

As highlighted in Figure 1 under “Things that make these children/youth feel worse,” the role of a child/youth’s social environment can positively or negatively influence their experience with meltdowns (e.g., how adults respond to meltdowns can result in a youth feeling embarrassed/ashamed). The children and youth in our study have provided a guide for neurotypical individuals on where to begin when providing support during meltdowns including understanding both individualized triggers and personal coping strategies, as well as responses that can exacerbate meltdowns (see Table 4). According to Lai and Szatmari (2019), the strategies identified here are aligned with those informed by a neurodiversity approach in that they consider extrinsic factors impacting the autistic individual and endeavor to improve the fit between the child and the environment. Based on the narratives shared by these youth and existing literature surrounding autistic perspective, adopting environmental adaptative approaches requires a shift toward a holistic framework with particular emphasis on social-ecological components.

### Implications for Research

This study (and the larger project it resides in) answers the call issued by Leadbitter et al. (2021) to conduct research relevant to autistic children and youth that embraces a neurodiversity framework. It also generates knowledge that can inform options for intervention that support positive coping strategies, personal agency, and wellness, which has been identified by Leadbitter et al. (2021) and the National Autistic Taskforce (2019) as crucial. Iemmi et al. (2017) outlined four core principles that are based on existing drawbacks in research literature as well as steps to move autism research forward. One of these steps is for future research to explore autistic experiences like BIMS, as experienced by autistic children and youth, including whether these phenomena interact with developmental and maturational processes. Through improved understanding, effective supports that are “autistic person-centered” can be generated for autistic children and youth experiencing BIMS (National Autistic Taskforce, 2019). Participatory and emancipatory approaches should be emphasized, particularly co-design approaches for the development of insider-informed supports.

### Limitations

A number of limitations should be considered for this study. The children and youth recruited for this study received diagnosis at a young age and are all connected with support services. There was also limited demographic diversity and intersection of multiple minority identities (e.g., socioeconomic status, race, ethnicity, gender, and sexuality) across our sample. Additionally, all the children and youth in this study have parents who believe that their children have valuable insight into their BIMS experience (and should therefore participate in this study), and who also value research. These aforementioned descriptors may explain why meltdowns were prominent in these children and youth’s lives as well as contribute to their understanding/experiences of BIMS. We also expect that this homogeneity across our sample has limited the breadth of experience we otherwise may have captured and described for this study. We did not have any child/youth participants who are non-speaking or who use augmentative communication, and so we have not captured potential variations in experience of this phenomena from a non-speaking perspective. This study would have benefited from additional follow up interviews with the youth to elicit more detailed descriptions and to ask more clarifying questions (Charmaz, 2006) regarding jargon, analogies and the content of the participants’ stories. While this analysis was inductive and matches Braun and Clarke’s (2020) description of a reflexive analysis in that we did not have *a priori* codes, we acknowledge that this analysis is deeply informed by our emerging understanding of BIMS phenomena, stemming from our previous work. The descriptions from these children and youth offer new ways to understand BIMS and the associated observable behaviors, and can be used to expand the thinking of educators, clinicians and parents. However, it is important to recognize that these narratives are unique to these youth and therefore, our descriptions should not be considered representative of the experiences of all members of the greater autistic community.

## Conclusion

The experience of burnout, inertia, meltdown, and shutdown are important parts of life for some autistic individuals, including children and youth. Children and youth may experience BIMS in ways that are different from autistic adults, and each child or youth has highly unique needs and preferences for support. The children and youth offer descriptions that contrast with clinical and academic approaches, which tend to focus on one component such as executive function, physiological state of arousal or social skills. Rather, they describe whole-person experiences, encompassing physical, cognitive and emotional components. This suggests that approaches to support/intervention should consider all of these elements as well, rather than taking a reductionistic or siloed approach. The children and youth in this study stress the importance of compassion and collaboration from adults who help them manage BIMS. Further research is required to develop an understanding of BIMS, especially with respect to how it is experienced by children and youth across diverse populations. Future research should leverage the insights and experiential knowledge of autistic children and youth to co-design support tool(s) for BIMS.

## Data Availability Statement

The datasets presented in this article are not readily available because this study is based on a small sample size and releasing generated datasets can potentially identify participants. Requests to access the datasets should be directed to CW, cwelch@hollandbloorview.ca.

## Ethics Statement

The studies involving human participants were reviewed and approved by Bloorview Research Institute Ethics Review Board Health Sciences Research Ethics Board at the University of Toronto. Written informed consent to participate in this study was provided by the participants’ legal guardian/next of kin.

## Author Contributions

JP conducted the thematic analysis of the data subset and co-wrote the manuscript. MP provided methodological guidance and support throughout all stages of research, provided suggestions and edits for manuscript. CP provided input and guidance from an autistic perspective, throughout all stages of research, provided suggestions and edits for manuscript. CW designed this study as well as the larger study, provided support throughout thematic analysis, and co-wrote manuscript. All authors contributed to the article and approved the submitted version.

## Conflict of Interest

MP has received consultation fees from Addis and Associates/Roche. She has received research funding from the Canadian Institutes of Health Research, the New Frontiers in Research Fund, and Autism Speaks. None of these funds received by MP were drawn upon for this study. The remaining authors declare that the research was conducted in the absence of any commercial or financial relationships that could be construed as a potential conflict of interest.

## Publisher’s Note

All claims expressed in this article are solely those of the authors and do not necessarily represent those of their affiliated organizations, or those of the publisher, the editors and the reviewers. Any product that may be evaluated in this article, or claim that may be made by its manufacturer, is not guaranteed or endorsed by the publisher.
